# Surface- and Tip-Enhanced Raman Spectroscopy as Operando Probes for Monitoring and Understanding Heterogeneous Catalysis

**DOI:** 10.1007/s10562-014-1420-4

**Published:** 2014-11-16

**Authors:** Clare E. Harvey, Bert M. Weckhuysen

**Affiliations:** Inorganic Chemistry and Catalysis group, Debye Institute for Nanomaterials Science, Utrecht University, Universiteitsweg 99, 3584 CG Utrecht, The Netherlands

**Keywords:** Heterogeneous catalysis, Raman, Scanning probe microscopy, Colloidal synthesis, Nanostructure, Nanotechnology

## Abstract

**Abstract:**

Surface-enhanced Raman spectroscopy (SERS) and tip-enhanced Raman spectroscopy (TERS) were until recently limited in their applicability to the majority of heterogeneous catalytic reactions. Recent developments begin to resolve the conflicting experimental requirements for SERS and TERS on the one hand, and heterogeneous catalysis on the other hand. This article discusses the development and use of SERS and TERS to study heterogeneous catalytic reactions, and the exciting possibilities that may now be within reach thanks to the latest technical developments. This will be illustrated with showcase examples from photo- and electrocatalysis.

**Graphical Abstract:**

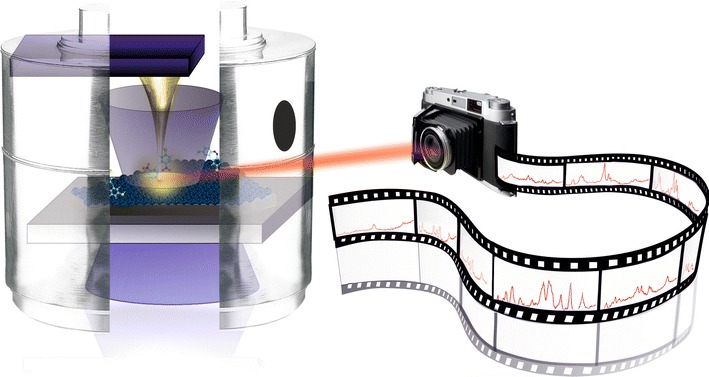

## Introduction

Surface-enhanced Raman scattering (SERS) is a phenomenon in which the Raman scattering intensity from molecules close to the surface of certain finely divided metals is amplified by several orders of magnitude [1]. As with ordinary Raman spectroscopy, SERS spectra show the molecular-vibration energies based on the frequency shift between the incident and scattered light by matter. The sensitivity enhancement of SERS in comparison to the conventional Raman process has resulted in more widespread applications, especially in surface chemistry where vibrational spectroscopic data reveal how molecules precisely interact with surfaces [1]. The analyte needs to be adsorbed onto the surface of the SERS substrate or very close to it (~10 nm), which can be challenging, however this mechanism has the added value of quenching the fluorescence from adsorbed molecules [2].

The unexpected enhancement of pyridine Raman signals on Ag electrodes was first reported by Fleischmann et al. in 1974 [3]. Surprisingly strong and potential dependent Raman signals from pyridine adsorbed on an electrochemically roughened Ag electrode have been observed. Subsequent investigations by Albrecht and Creighton [4] and Jeanmaire and Van Duyne [5] in 1977, led to the discovery of SERS and its remarkable enhancement effects. It was proposed by Van Duyne and co-workers that the enhancement of the Raman scattering was due to an increase in the electromagnetic field at the roughened surface. Creighten et al. hypothesised that resonance Raman scattering was responsible due to the creation of a charge-transfer absorption band between the adsorbate and the surface. These two ideas, now known as the *electromagnetic* and *chemical enhancement* mechanisms, have dominated the mechanistic debate in the SERS community for the past 30 years, and most probably will continue to do so.

The recent revival of interest in SERS as an interesting and valuable approach to probe heterogeneous catalytic reactions has incited a flurry of publications on the subject. Possibly the most interesting reports now deal with the developments leading to the use chemically inert SERS probes [6, 7] as well as to methods for anchoring SERS-active nanoparticles on the external or internal surface of catalyst supports and molecular sieves [8, 9]. They represent exciting new steps on the road towards the wide-ranging use of SERS as an *operando* probe for monitoring and understanding heterogeneous catalysis.

In this article we discuss the use, recent developments and potential of SERS and TERS in the field of heterogeneous catalysis. Special attention will be focused on some historic developments, recent applications for e.g. reaction monitoring and the technical requirements to be further explored to make this methodology a real asset in the ever-expanding toolbox of in situ spectroscopic methods for heterogeneous catalysis research. The goal of this paper is, however, not to give a comprehensive overview of the entire fast-growing research field. Within this context, we refer the reader to some older and more recent review papers on this topic to put the described developments in the proper context [10–12].

## A Brief History to SERS of Heterogeneous Catalysis

### 1981–1986: First Applications of SERS in the Field of Heterogeneous Catalysis

The first steps towards the use of SERS in the field of heterogeneous catalysis were taken in the 1980s with the study of adsorbates at catalytically relevant interfaces by the Dorain group at Yale University. Initial work was done on Ag powders with a rough surface on the nanoscale, on which surface species such as SO_3_^2−^ and SO_4_^2−^ were observed and identified [13–16]. These early forays into kinetic studies utilising the SERS methodology were tentative in their conclusions, as it was already noted early on that the intensities of SERS peaks are dependent on the metal surface roughness as well as on the adsorbate concentration. The SERS-active phase on the Ag powder was formed in situ in the case of the reactions of NO_2_/N_2_O_4_ with Ag. SEM measurements were performed to confirm the theory that Ag microstructures were formed on the surface of the Ag powder. It was confirmed that the formation of these microstructures was due to the surface layer of Ag_2_O reacting with the initial pulse of the gas to form AgNO_3_ + Ag + NO. The Ag atoms formed then migrated freely due to the thermal energy released by the reaction, forming Ag microstructures. These first series of studies demonstrated the potential of SERS for studying heterogeneous catalysis, as well as highlighting the challenges of separating the catalytic reaction from the SERS analysis.

### 1987–1993: Introduction of SERS in the Field of Electro-catalysis

Following the first reported successes of SERS for heterogeneous catalysis research it could have been expected that the field of ultra-high vacuum (UHV) surface science would take on the challenge of SERS, but this was not really the case. The early recognition that the SERS effect was limited to roughened surfaces of ‘coinage’ metals most probably dampened the interest of the UHV surface-science community. The concurrent emergence of electron energy loss spectroscopy (EELS) and infrared-reflection absorption spectroscopy (IRAS) for UHV-based systems most probably encouraged this indifference, as unlike SERS these vibrational techniques were applicable to the ordered mono-crystalline metal surfaces that were at least in the early days the primary focus of the UHV community. The markedly greater interest shown by the electrochemical community in SERS was probably due in part to the near-exclusive usage (at that time) of polycrystalline metal electrodes, along with the straightforward preparation of SERS-active surfaces by means of controlled-potential oxidation–reduction cycles.

It is important to mention here that the group of Michael Weaver at Purdue University took a new approach to SERS substrates in the late 1980s by coating Au electrodes with thin layers (3–4 monolayer) of transition metals, such as Ru, Rh and Pt [17]. These thin metal layers provided the catalytic surface, whilst the Au electrode beneath enhanced the Raman scattering.

The transition metal thin films were electrodeposited onto an electrochemically roughened gold electrode. The thickness of these transition metal layers can be controlled by varying the potential across the electrode. By utilising transition metals in this way, a much broader range of catalytic reactions were now available for study. The Weaver group investigated catalytic systems ranging from CO and SO_2_ oxidation to methanol and formic acid oxidation [18–21].

An early example of these studies is the electro-oxidation of CO over Rh and Ru coated Au electrodes, followed by the observation of SERS bands in the C–O stretching region, which disappeared upon voltage increase at the electrode [22]. Corresponding cyclic voltammetry measurements show CO electro-oxidation occurring at the same voltage at which the SERS bands disappear, confirming the reaction. However, in 1988 it still took 10 min to collect these SERS spectra, preventing practical kinetic measurements.

**Fig. 1:**
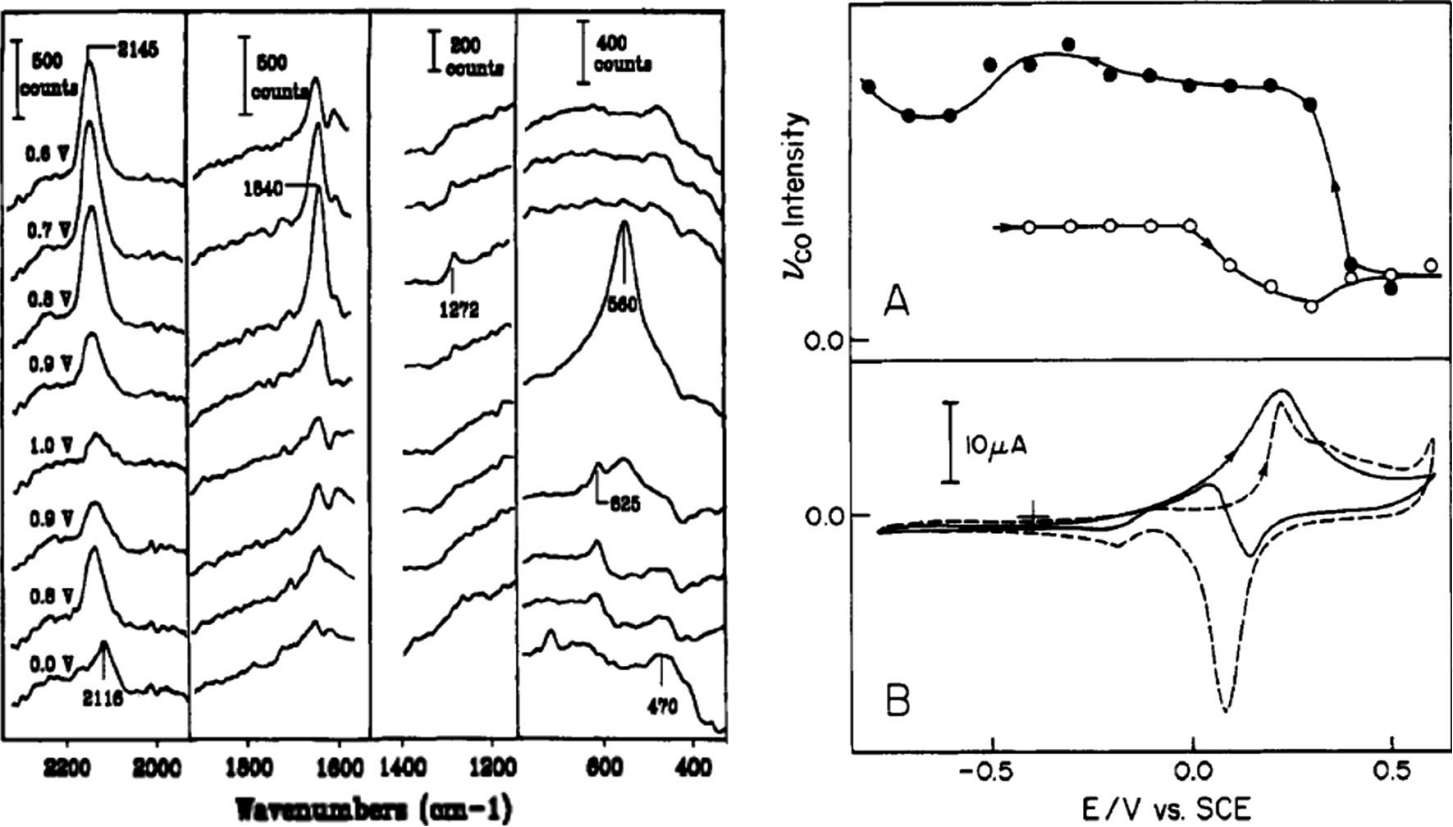
Potential-dependent SER spectral sequence obtained on gold during voltammogram at 10 mV s^−1^ for 0.5 mM methanol in 0.1 M HClO_4_ from −0.3 to 1.5 V versus SCE and return (*left*). Intensity of ν_CO_ band (arbitrary scale) versus electrode potential extracted from SER spectra on gold in 0.1 M KOH with 0.1 M methanol, during 10 mV s^−1^ potential sweep from −0.8 to 0.6 V and return. The *open* and *filled circles* refer to the forward and reverse sweeps, respectively (*right*). Corresponding voltammograms obtained in presence (*solid trace*) and absence (*dashed trace*) of 0.1 M methanol. Reproduced with permission from Ref. [21]. Copyright American Chemical Society, 1993

A significant discovery enabled through the use of in situ SERS is exemplified in the case of methanol oxidation, SERS spectra and cyclic voltammograms of which are shown in (Fig. 1) [21]. It was thought that methanol oxidation over Au was not active because the intermediates were not formed on the Au surface, thus preventing reaction. However, the SERS results clearly showed that the required formate intermediates were indeed formed on the Au surface. This demonstrated that the molecular fragmentation of methanol, though necessary, was not in itself sufficient, and that it was in fact the availability of oxygen co-adsorbates that was the controlling factor in methanol oxidation.

### 1992–1999: Moving Beyond the Field of Electro-catalysis

An important limitation of the transition metal thin films approach developed and used by Weaver and co-workers in the late 1980s and early 1990s was the presence of residual ‘pinhole’ sites that exposed the underlying Au substrate. However, this complication turned out to be much less of an impediment to the utilization of the technique in gas-phase environments, particularly at the elevated temperatures of catalytic relevance, since Au displays near-negligible adsorptive properties under these conditions. It was found that the Pt-, Rh- and Ru- coated Au surfaces exhibited excellent temporal stability when under ambient pressure gas-phase environments, which allowed the exploration of the utility of the SERS approach for probing the adsorption of molecules at such catalytic relevant surfaces [23].

The advent of CCD technology enabled spectra to be obtained at significantly faster rates (1–2 s). This technical advance also lead to the improved sensitivity in Raman measurements, as demonstrated by McCreery and co-workers detecting adsorbates at the carbon-aqueous interfaces even in the absence of SERS, [24] and the Tian group obtaining Raman spectra for a diverse range of adsorbates at the transition metal-solution interface in the presence of only mild surface enhancements, more specifically ~30–100 [25, 26].

The Weaver research group built upon their experience in the SERS field to move from electrochemical cells to gas-flow reaction systems. The combination of in situ SERS and in-line mass spectrometry analysis of the gas flow enabled them to investigate a range of chemical reactions. The changing intensities of Raman bands of surface species and simultaneous analysis of the reaction products enabled new physicochemical insight into the reaction kinetics and related mechanisms over transition metal surfaces.

**Fig. 2:**
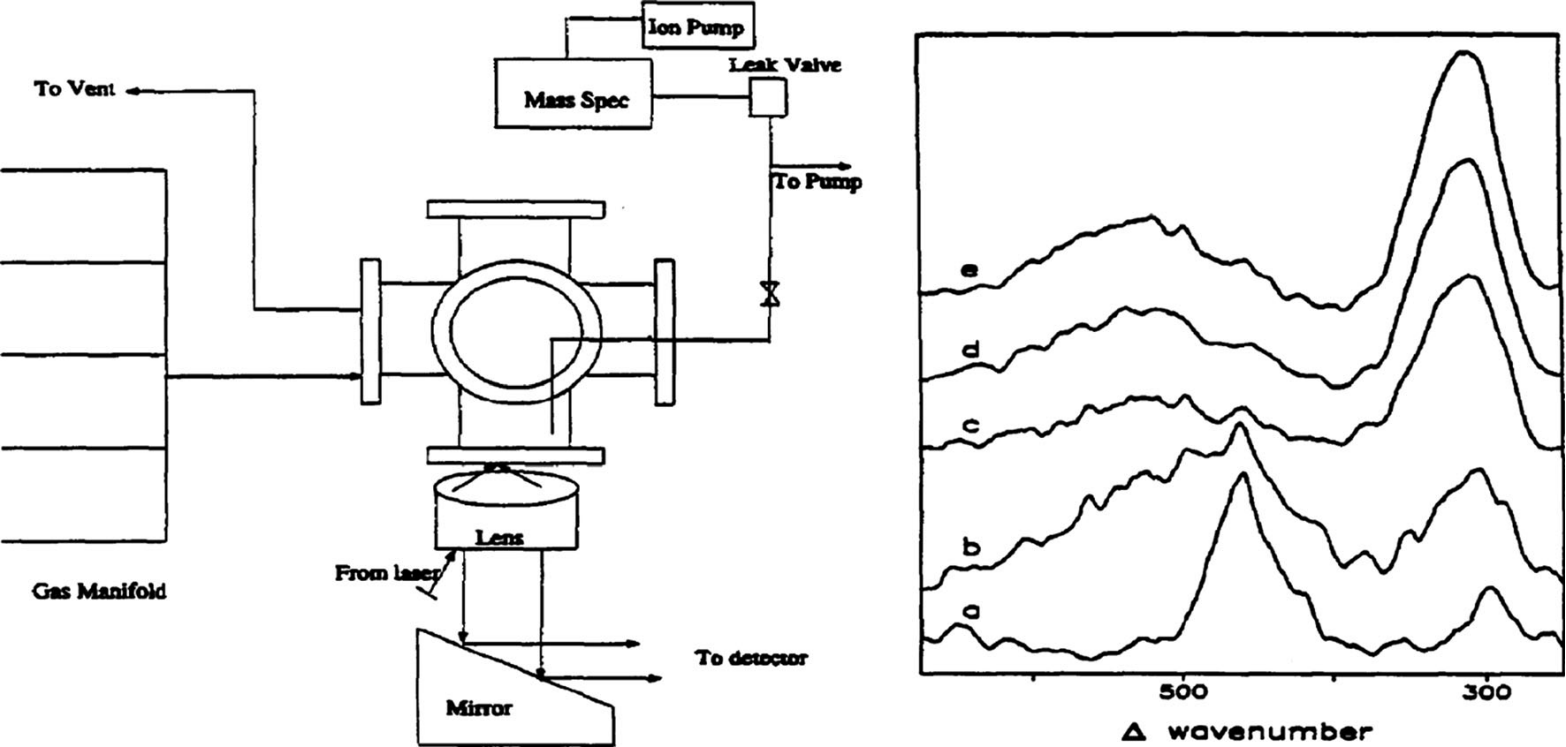
(*left*) Schematic of the SERS gas-flow set-up; and (*right*) time-resolved SER spectra for a reduced Rh surface exposed to NO. The surface was first treated with CO at room temperature, evacuated, and then held in 80 ml/min Ar. NO at 10 ml/min was then added to the gas feed. Spectra shown are for times of *a* 0, *b* 30, *c* 60, *d* 120, and *e* 240 s after the NO flow was initiated. Reproduced with permission from Ref. [27]. Copyright Elsevier, 1996

The catalyst systems investigated include the in situ SERS on the stability and reactivity of adsorbates, including SO_2_, NO, CO, H_2_, O_2_ and methanol, up to temperatures of 500 °C. A schematic of the set-up, along with a selection of SERS spectra for a reduced Rh surface in contact with NO are shown in Fig. 2. More specifically, the initial work in the gas flow system established the adsorption spectra of M–NO, M–Co and M–O, as well as their stability at temperatures of up to 350 °C [28]. This was followed by work in which intermediate cyanide surface species were spectroscopically observed at temperatures above 185 °C during the oxidation of CO by NO over Rh-coated Au surfaces [29]. The further use of on-line mass spectrometry to detect the reaction products during temperature ramps and gas-flow composition elucidated even more information [30]. Using their knowledge of these methods, the Weaver group went on to study the surface species and products of the NO–H_2_ reaction on Rh, the CO–NO reaction of Pt and Pd surfaces, as well as the methanol oxidation on Rh surfaces [27, 31–36]. Also studied were the metal oxides of the Pt group, their formation as a function of temperature, and their reduction kinetics and thermal oxidation [37, 38].

## Heterogeneous Catalysis and SERS: Developments in the Last Decade

Single-molecule SERS (SM-SERS) was first demonstrated in 1997, garnering new interest in SERS from a much wider audience. Advances in nanotechnology and synthetic methods have also assisted in this new wave of research, leading to several significant advances in the field.

### Revival of SERS in the Field of Electro-Catalysis

Following a 5-year hiatus, a revival of interest in the use of electrochemical SERS around the turn of the millennium led to an expansion of the scientific community involved in the study of SERS of heterogeneous catalysis.

**Fig. 3:**
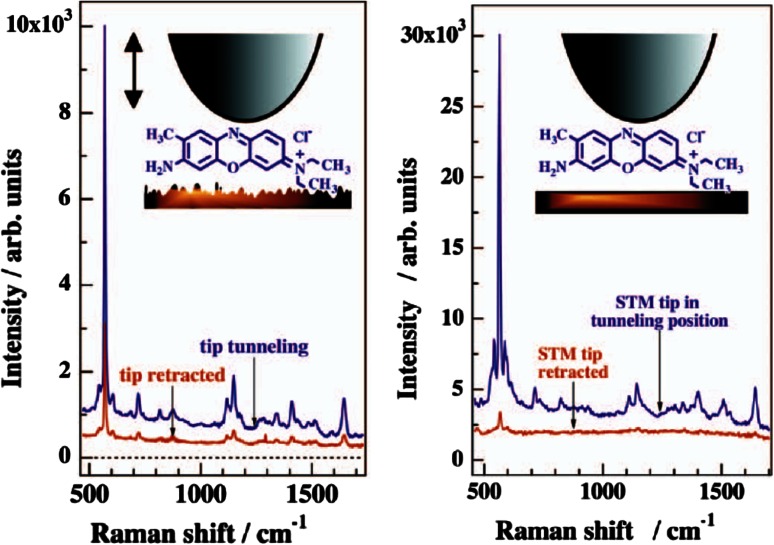
(*left*) SERS and TERS for Brilliant *Cresyl Blue* (BCB) on a mildly activated Au film; and (*right*) Resonance Raman Scattering (RRS) and TERS for BCB at a smooth Au film with a thickness of 12 nm. Reproduced with permission from Ref. [42]. Copyright Wiley–VCH, 2002

It is worth noting that many SERS studies have been undertaken on surface chemistry and molecules adsorbed at metal electrodes. These are often closely related to the catalytic reactions discussed in this paper, however to discuss these fully is beyond the scope of this work. Some noteworthy examples include the seminal SERS and TERS work of Ertl and Pettinger, an example of which is showcased in Fig. 3 [39–42].

The Weaver group returned to SERS studies of electro-catalysis after several years of focussing on utilising SERS for gas-phase reactions, publishing a couple of new electro-catalytic SERS articles in the midst of more fundamental electrochemical work [43–45]. The death of Weaver in 2002 clearly left a vacuum in the fields of electrochemistry and SERS that was later on filled by several new research groups. In this respect, it is worthwhile to mention that a special edition of *The Journal of Electroanalytical Chemistry* was dedicated to the memory of Michael, to which Ertl and co-workers, as well as Koper et al., contributed papers on SERS in the field of electro-catalysis [46, 47].

**Fig. 4:**
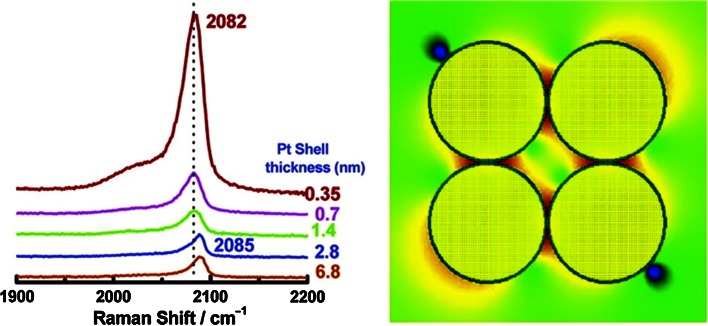
(*right*) SER spectra of CO adsorbed on 55 nm sized Pt-coated Au nanoparticles with different Pt shell thicknesses in CO-saturated 0.1 M HClO_4_ at 0.0 V; and (*left*) 3D-FDTD simulated electric field distribution for a 1.5 nm Pt layer surrounding a 55 nm Au nanoparticle. Reproduced with permission from Ref. [56]. Copyright American Chemical Society, 2006

In 1989 Zhong-Qun Tian was appointed at Xiamen State University, and focused his research on electrochemical SERS and its application in the field of heterogeneous catalysis. This research group revisited and made important improvements upon the older electro-catalytic work of the Weaver group [48–52], progressively making their own way in the field first with a new electrochemical cell with heating capabilities, [53] and then later on working by working with Ag electrodes. [54, 55] In 2006 the method for Pt-shell Au-core nanoparticles was developed as an alternative to transition metal-coated electrodes. [56, 57] This important advance allowed researchers to utilise the high SERS intensity formed at hotspots between nanoparticles without exposing the Au nanoparticle surface to the chemical reaction, essentially decoupling the catalysis and sensing/enhancing properties of the metal surface. This is illustrated in Fig. 4 for CO adsorption on Au nanoparticles surrounded by a Pt shell of various thickness.

A new method for coating SERS-active electrodes with a monolayer of small Pt or Pd nanoparticles was developed in 2005 by the Perez group in Alicante [58–61].The nanoparticles coating of the electrode are small enough (i.e, 4 nm) that the SERS enhancement is maintained, whilst the use of a layer of nanoparticles rather than an ultrathin layer of Pt metal over the electrode gives an improvement in stability. A combined SERS and AFM study was undertaken by Scheijen et al. in 2007, looking at the influence of the Pt nanoparticles deposition procedure over Au electrodes on the electro-oxidation of small organic molecules. These authors concluded that the method of nanoparticles deposition has a strong influence on the size and homogeneity of the nanoparticles, and the resulting catalytic and SERS activity [62].

Studies of the electrochemical reduction of keto-esters over Pt surfaces have been undertaken by Attard et al. at Cardiff University, using the previously mentioned Tian method of coating Au nanoparticles with Pt. Mechanistic insight into these chemical reactions show the potential of the SERS methodology for measuring increasingly complex catalytic systems [63, 64]. The electrochemical SERS work of Koper and co-workers at Leiden University also utilizes a variety of bare and transition metals-coated electrodes, in combination with which SERS is used to gain a deeper understanding of the systems involved in solar fuels production [47, 62, 65, 66]. Along the same line of research, recent publications of the Bell group at UC Berkeley report on the use of cobalt oxide- and nickel oxide-coated Au electrodes to follow the electrochemical evolution of O_2_ [67, 68].

### Applications of Anchored SERS Nanoparticles

Moving towards the implementation of the SERS methodology in more industrially relevant catalytic systems, the SERS substrates are required to be physically robust as well as sufficiently Raman signal enhancing. Various methodologies for anchoring SERS nanoparticles to support materials have been reported, which look promising for future use in heterogeneous catalysis. Some highlights on these approaches are discussed below.

In 2004 a core–shell Au–Pt nanoparticle film was fabricated by a self-assembly method on a silicon wafer, and its application as a catalyst and as SERS substrates was investigated [69]. The nanostructured film exhibited high catalytic activity and SERS, demonstrating its potential use in heterogeneous catalysis and as a SERS substrate. In addition, it should be possible to tune the surface properties of the film by controlling the size, composition and surface properties of core–shell nanoparticles. The self-assembly method leads to a highly reproducible substrate, with great potential for sensing applications in catalysis and other fields.

**Fig. 5:**
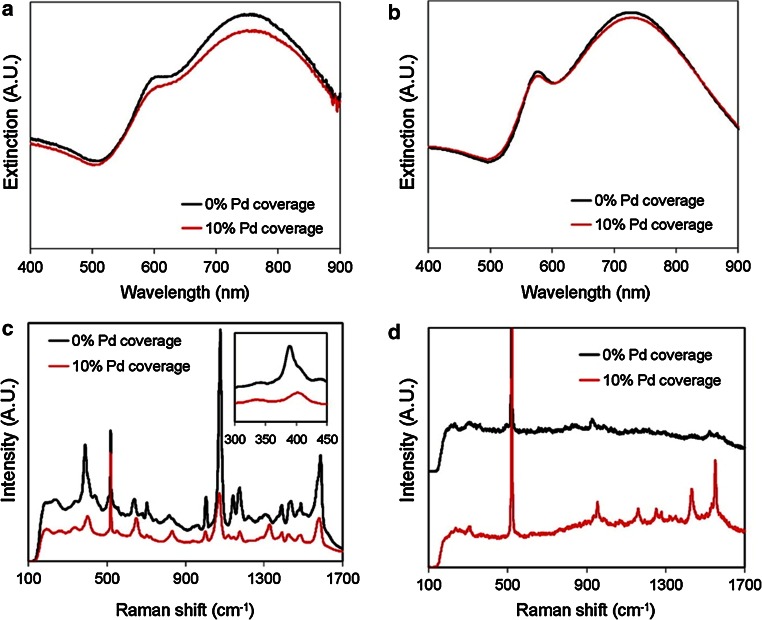
**a** UV–Vis spectra of Au nanoparticles and Pd/Au nanoparticles with 10 % Pd coverage; **b** calculated spectra of Au and Pd/Au nanoparticles; **c** Raman spectra of pMA chemisorbed on Au nanoparticles and Pd/Au nanoparticles with 10 % Pd coverage with the *inset* illustrating the metal-S stretching region; and **d** Raman spectra of 1,1-DCE in H_2_O (50.9 µM) on Au nanoparticles and Pd/Au nanoparticles with 10 % Pd coverage. Reproduced with permission from Ref. [70]. Copyright American Chemical Society 2008

Heck et al. reported that sub-monolayer coverage of Pd on Au nanoparticles showed unexpected high activity for the aqueous-phase hydrodechlorination of trichloroethene [70]. This nano-shell SERS sensing approach was combined with sub-monolayer Pd deposition to combine the catalytic and SERS-active functionalities, enabling the operando monitoring of catalytic activity. The nanoparticles were immobilised on Si wafers and plasma-cleaned prior to adsorption and reaction experiments in the aqueous phase. UV–Vis and SERS spectra of the uncoated and Pd-coated Au shells are shown in Fig. 5. Chemical reactions of the adsorbate species were observed as they proceeded on the catalyst surface with increasing reaction time, enabling the detection and identification of reaction intermediates. The authors express their hope that this showcase example of nano-shell may further the SERS approach to gather new mechanistic insights into other liquid-phase chemical reactions.

**Fig. 6:**
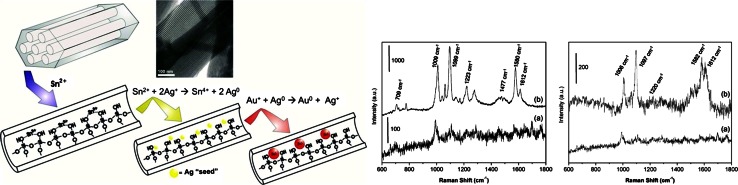
Schematic of the synthesis of Au nanoparticles within the pores of SBA-15 (Au/SBA-15) (*top*) by sequential redox and galvanic reactions through an intermediate product, Ag/SBA-15; (*bottom left*) (*a*) Raman spectrum of 1 × 10^−2^ M 4-Mpy that was drop-casted and dried on a CH_3_-capped SBA-15 sample, and (*b*) SERS spectrum of 1 × 10^−5^ M 4-Mpy that was drop-casted and dried over Au/SBA-15, which was prepared in the presence of CTAB; (*bottom right*) (*a*) Raman spectrum of 1 × 10^−2^ M 4-Mpy dried over SBA-15, and (*b*) SERS spectrum of 1 × 10^−5^ M 4-Mpy dried over Au/SBA-15. Reproduced with permission from [8]. Copyright American Chemical Society, 2011

Another promising approach makes use of mesoporous silica. Mesoporous silicas are a well- known class of catalyst supports that have large pore sizes and volumes as well as well-ordered structures. In 2011, Silva et al. demonstrated SERS enhancement from Au nanoparticles within the mesoporous channel pores of SBA-15 [8]. An overview of the ion-exchange synthesis method is shown in Fig. 6, along with two examples of SERS spectra obtained using the Au/SBA-15 material. Using 4-mercaptopyridine (4-Mpy) as the SERS reporter molecule, SERS enhancement factors as high as 10^5^ could be obtained. The high SERS enhancement given by the Au/SBA-15 as compared to any other powered SERS substrates appears to be the result of the formation of SERS “hot spots” due to the side-by-side alignment of Au nanoparticles within the cylindrical channel pores of the mesoporous silica host. Because of their powdered forms, longevity and high SERS enhancement factors, the Au/SBA-15 material can be expected to serve as a simple substrate for SERS-based analysis of various analytes, including reactants and reaction products during heterogeneous catalysis. The potential for the material has already been recognised, with a new, simplified method of preparing Au nanoparticles inside the SBA-15 channels recently published [71].

A recent promising nanomaterial is graphene, and has been recently demonstrated as a support to disperse and stabilize various metal and metal oxide nanoparticles. Huang et al. describe the formation of Au nanoparticle-graphene oxide (Au–GO) and Au nanoparticle-reduced GO (Au-rGO) composites [72]. These composite materials were used to demonstrate SERS of the molecule p-aminothiophenole (pATP) as well as of the catalytic reduction of *o*-nitro-aniline. The impressive results for both applications due to the electronic interaction of the graphene with the Au nanoparticles, show great promise for the possible combination of SERS and catalysis over graphene substrates, and have already inspired several further investigations into the use of graphene oxide in combination with SERS [73–75].

In 2012, different shapes of Pt nanoparticles were used to enable the monitoring of a surface reaction on different surface sites. Spherical and octahedral–tetrahedral Pt nanoparticles were used with the (111) surface orientation of the octahedral–tetrahedral nanoparticles facilitating the reduction of acetaldehyde oxime into ethylamine. This reduction process was not observed over spherical nanoparticles, demonstrating the combination of SERS-active metals and the use of preferentially ordered nanoparticles allows SERS studies of structure-sensitive surface reactions. [76]

### Monitoring Chemical Reactions Directly in Colloidal Suspension

**Fig. 7:**
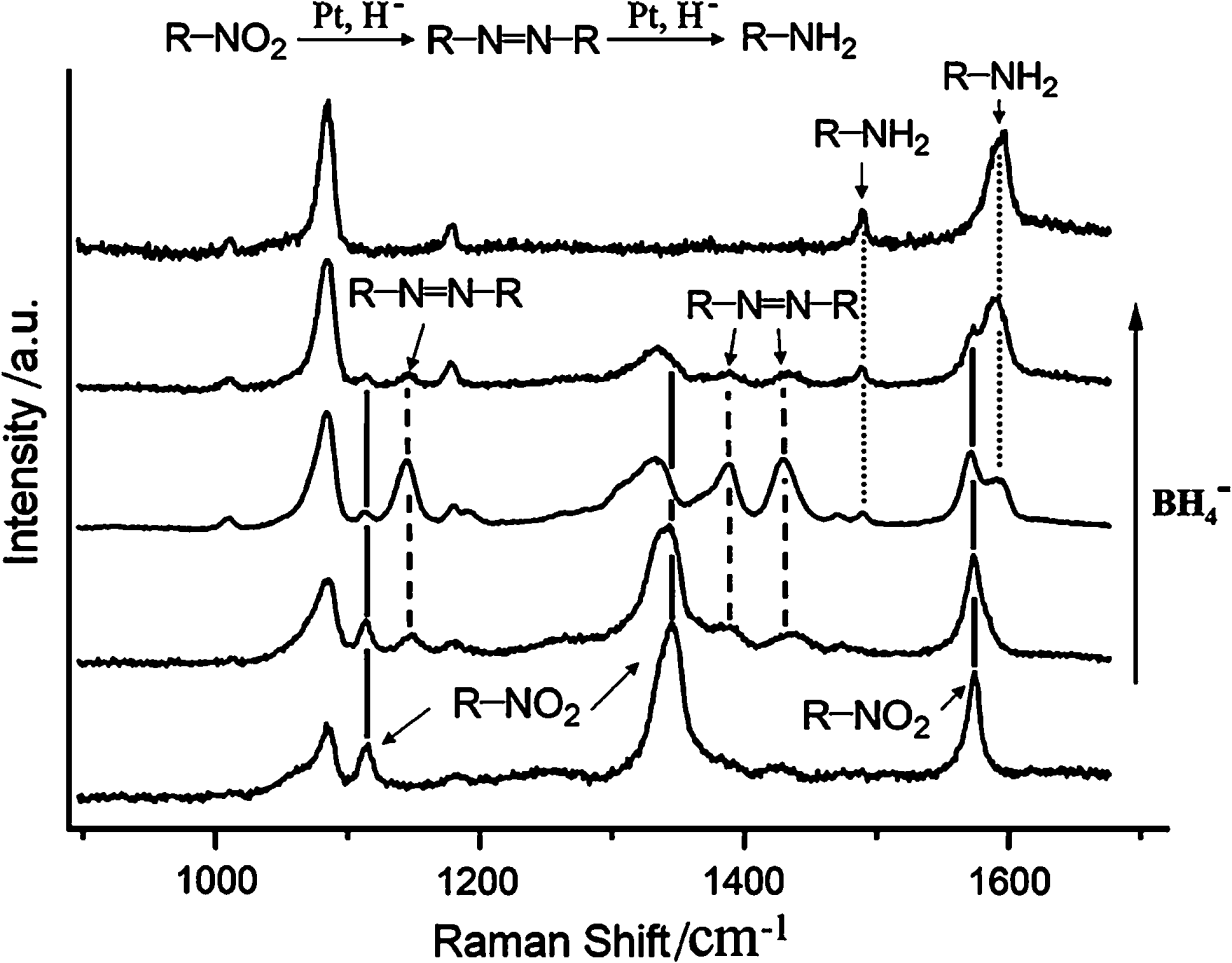
SER spectra recorded during the Pt-catalyzed hydride reduction of an aromatic nitro compound using different amounts of the reducing reagent NaBH_4_. From *bottom to top*: increasing volumes of 10 mM NaBH_4_ solution were added to 4-NTP-functionalized Au/Pt/Au core/shell nanoraspberries. Reproduced with permission from Ref. [77]. Copyright American Chemical Society 2011

The Schlücker group of the University of Osnabruck demonstrated in 2011 the successful synthesis of so-called bifunctional Au/Pt/Au nanoraspberries [77]. These peculiar nanoparticles successfully integrated the SERS activity of Au nanoparticles with the catalytic activity of the Pt surface, whilst enabling the monitoring of chemical reactions directly in colloidal suspension. This is illustrated in Fig. 7. They also presented a multivariate approach for the quantitative analysis of in situ SERS spectra that allows to determine the chemical identity of the involved molecular species and the quantification of their relative contributions, which is a requirement for establishing reaction mechanisms and testing the related kinetic models. Finally, Schlücker et al. noted that the presented approach of using bifunctional Au/Pt/Au nanoraspberries for SERS monitoring of Pt-catalyzed reactions is currently limited to molecular species containing a surface-seeking group in order to experience the necessary SERS enhancement.

Joseph et al. have also recently used the idea of small catalytic nanoparticles supported on large SERS nanoparticles, following the catalytic reduction of pNTP with NaBH_4_ over Pd nanoparticles of 2 nm in size supported on 40 nm-sized Au nanoparticles. [78]

More recent work by the Schlücker group has built upon the principle of small catalytically active nanoparticles supported on larger, SERS active nanoparticles [79]. The 80 nm Au core is encapsulated within a thin silica shell of ~1.5 nm in size, then thiol groups are used to attach 5 nm Au nanoparticles to the surface. These bimetallic particles are used to monitor the Au-catalysed reduction of pNTP in colloidal suspension, whilst the inert silica shell protects the Au metal core surface from direct contact with the chemical species and prevents unwanted photocatalytic side reactions.

Other Au and Ag nanostructures have been synthesised and tested for both SERS and catalytic activity, though not in combination. One such example is the three-dimensional dendritic Au nanostructures developed by Huang et al. [80]. The high surface-to-volume ratio of the dendritic structures led to high catalytic efficiency, and the structure provided multiple sharp corners and edges for SERS hotspots. However, the adaption of such substrates for use in catalytic conditions could be complicated, and is clearly a challenge for future research.

### SERS and Plasmon-Driven Catalysis

For over a decade, pATP has been regarded as a prototypical probe for assessing the nature of SERS measurements [81]. As pATP interacts strongly with Ag and Au, gives strong SERS signals, and has been regarded as a model compound for probing the chemical enhancement of SERS due to its potential and wavelength dependent spectra [82]. However, difficulties in reproducing the SERS spectra of pATP alone led to further investigations, through which two different explanations were derived. Sun and co-workers first hypothesised that the catalytic conversion of pATP to dimercaptoazobenzene (DMAB) accounted for the unexplained SERS spectra in 2009 [83]. This conclusion was supported by further theoretical and experimental work by Sun and co-workers, who then also concluded that DMAB could be produced by 4-nitrobenzenethiol (4-NBT) [81, 84, 85]. It has been found that these plasmon-driven reactions are strongly dependent on substrate, wavelength and time. More significantly, these instances of plasmon-driven catalysis have shown that SERS can be an invasive technique under certain conditions, and that the species measured may not be the original surface species [86]. In contrast, the work of Kim et al. supports the hypothesis that the SERS bands under debate are in fact a result of the chemical enhancement mechanism [87–89].Within this context it is important to refer to a recent study of Kang et al. [90] of the Harbin Institute of Technology, who have monitored the plasmon-driven conversion of pNTP into DMAB using a single Ag particle of 2 μm in size and having a roughened surface. This detailed SERS study provided laser wavelength- and power-dependent conversion rates for the reduction of pNTP into DMAB.

**Fig. 8:**
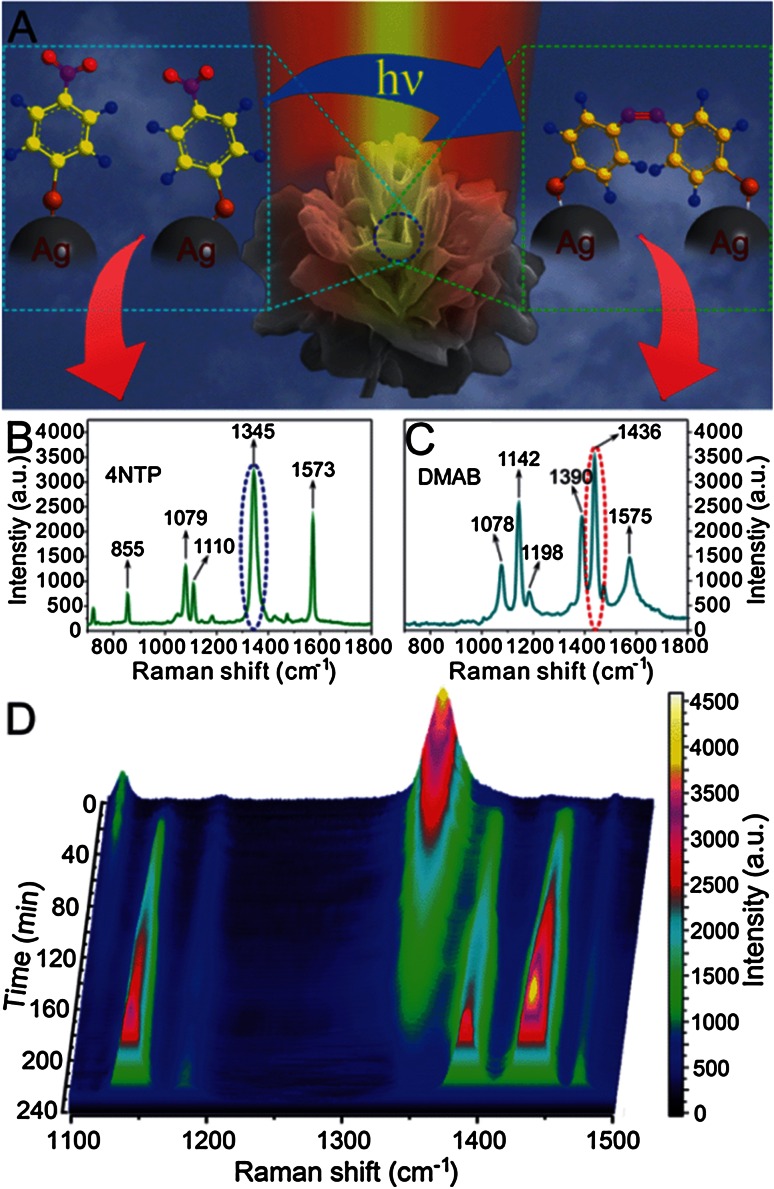
**a** Schematic of the plasmon-driven surface catalysed reaction of pNTP dimerizing into DMAB monitored by a single particle SERS substrate, more specifically consisting of hierarchical peony-like Ag microflowers. **b**,**c** SERS spectra of pNTP and DMAB. **d** *Color-coded* intensity of spatiotemporal SERS mapping under continuous 632.8 nm laser excitation. Reproduced with permission from [91]. Copyright Royal Society of Chemistry, 2014

As a last example to illustrate the concept of plasmon-driven catalysis we highlight recent research work of Tang and co-workers of the Chinese Academy of Sciences in Hefei. This group has developed SERS-active Ag particles consisting of hierarchical peony-like microflowers with a highly roughened surface, as illustrated in Fig. 8a. By making use of these constructs in combination with the dimerization reaction of para-nitrothiopenol (pNTP) into DMAB is was possible to determine the detailed kinetics of this process as a function of the different morphologies of the microflowers synthesized. Fully developed nanostructures not only showed a larger SERS enhancement, they also lead to an increase in the reaction rate of the dimerization process [91].

## Challenges Imposed on SERS for Performing Operando Spectroscopy Measurements

The true aim for the SERS methodology in catalysis research is of course to perform *operando* measurements, leading to new fundamental insights into catalytic intermediates and related transition states. In order to achieve this ambitious goal, further advances must be made to existing SERS probes, enabling the use of SERS under the often extreme conditions encountered inside a catalytic reactor; i.e., at high temperatures and pressures. The most important challenges that are yet to be fully overcome are chemical stability, thermal stability, spectral reproducibility and data analysis. These challenges will now be discussed in more detail below.

### Chemical Stability

One of the primary considerations in the use of SERS for label-free monitoring of catalytic reactions is the exposure of a chemical reaction to an additional transition metal surface in the form of SERS nanoparticles or a planar SERS substrate.

For true operando measurements that are unlimited by the catalyst surface being Au or Ag, SERS surfaces with a high level of chemical stability must be developed, or adapted from existing particles or surfaces. One solution would be to use a SERS surface that could be coated with e.g. Al_2_O_3_, however under true reaction conditions it would be preferable to have inert particles that could be distributed within the catalyst material, being preferentially within its shaped form (i.e. catalyst body), without causing any transport limitations or disruption to the gas flow in the catalyst bed.

Previous work in the field of spectroscopy has seen attempts to modify the surface specificity of SERS nanoparticles by various methods. One of the oldest of these approaches is the functionalisation of the nanoparticle surface with alkane thiols, for example, to enable SERS measurements of neutral molecules that would not usually interact with the charged capping of a SERS active nanoparticle [11]. Another method to bring SERS and heterogeneous catalysis together is the use of an ultra-thin shell of other transition metals, such as Pt and Pd [17]. These systems effectively ‘borrow’ the SERS enhancement from the Au core nanoparticles, whilst presenting a catalytically active transition metal outer surface. Until recently, the destabilisation of the nanoparticles upon the removal of the stabilising coating layer has prevented these forms of SERS substrates from application beyond electrochemical systems, though the work of the Schlücker group has begun to address this important topic [79].

**Fig. 9:**
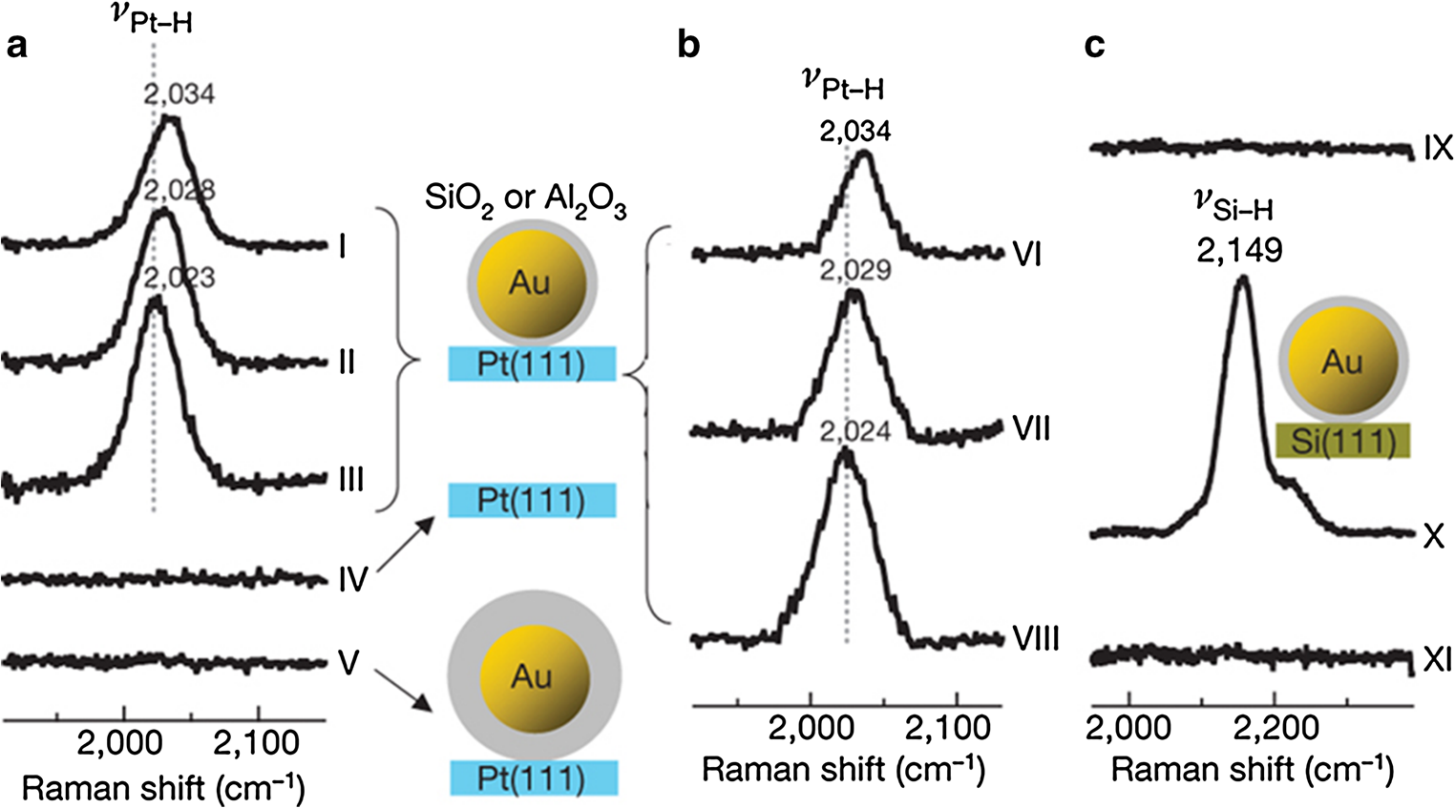
**a** Potential-dependent SHINERS spectra of hydrogen adsorbed on a Pt(111) surface. *Curve I*, at −1.2  V; *curve II*, −1.6  V; *curve III*, −1.9  V; *curve IV*, without Au/SiO_2_ nanoparticles; *curve V*, with the thicker shell nanoparticles at −1.9  V; **b** potential-dependent SHINERS spectra of hydrogen adsorbed on a Pt(111) surface using Au/Al_2_O_3_ nanoparticles. *Curve VI*, −1.2  V; *curve VII*, −1.6  V; *curve VIII*, −1.9  V; (c) SHINERS spectra on Si(111) wafer treated with 98 % sulphuric acid (*curve IX*), 30 % HF solution (*curve X*) and O_2_ plasma (*curve XI*). Reproduced with permission from Ref. [6]. Copyright Nature Publishing Group 2010

Shell isolated nanoparticle-enhanced Raman spectroscopy (SHINERS) is a powerful SERS method first proposed in 2010 by the group of Tian et al [6]. It works under the premise that the formation of an ultrathin silica shell around a SERS nanoparticle will prevent the direct chemical interaction of an analyte with the nanoparticle, whilst being thin enough to still be within the range of the electromagnetic enhancement mechanism of SERS. The silica shell also prevents the nanostructure of the nanoparticle from degrading on an atomic level, therefore making the SERS signal more stable. The sensitivity of this innovative method to the silica shell thickness is illustrated in Fig. 9.

The challenge of the interference of a SERS substrate in a particular system is also a hot topic within e.g. the bio-analysis field, where it has been noted that the exposed metal surface of metal nanoparticles can easily adsorb interfering molecules in the biological environment, leading to variations of the original SERS signals as well as possible biotoxicity [92]. Common solutions include the use of a biomolecule surface-coating, such as bovine serum albumine (BVA), non-toxic polymer coatings, liposome coatings and silica coatings. Such approaches could also inspire the field of heterogeneous catalysis.

### Thermal Stability

Chemical reactions often run well over 300 °C, so it must be possible to use SERS under such conditions. The thermal stability of nanoparticles is a well-known topic in heterogeneous catalysis, with the sintering of nanoparticles at high temperatures being a major field of study. One commonly used solution in heterogeneous catalysis is the synthesis of active nanoparticles within a confined micro- or nanoporous system, which restricts the mobility of the nanoparticles under heating, and so prevents sintering. As discussed above, Silvia et al. utilized a similar concept for their SBA-15 crystals containing Au nanoparticles, though they did not extend their study to look at thermal effects [8]. An investigation into the heating effects on nanoparticle films by Kho et al. confirmed that lateral particle diffusion as a result of heating results in a loss of SERS signal [93]. Other studies focusing on roughened metal substrates have observed some effect of heating on the SERS activity, though the changes are reversible upon cooling [94–96].

As discussed previously, high-temperature SERS has been achieved with the use of electrochemically-roughened surfaces as SERS substrates. These transition metal surfaces demonstrate a much higher degree of thermal stability than the metal nanoparticles more frequently employed in recent years, though they do not have the extremely high SERS enhancement factors often seen with metal nanoparticles.

**Fig. 10:**
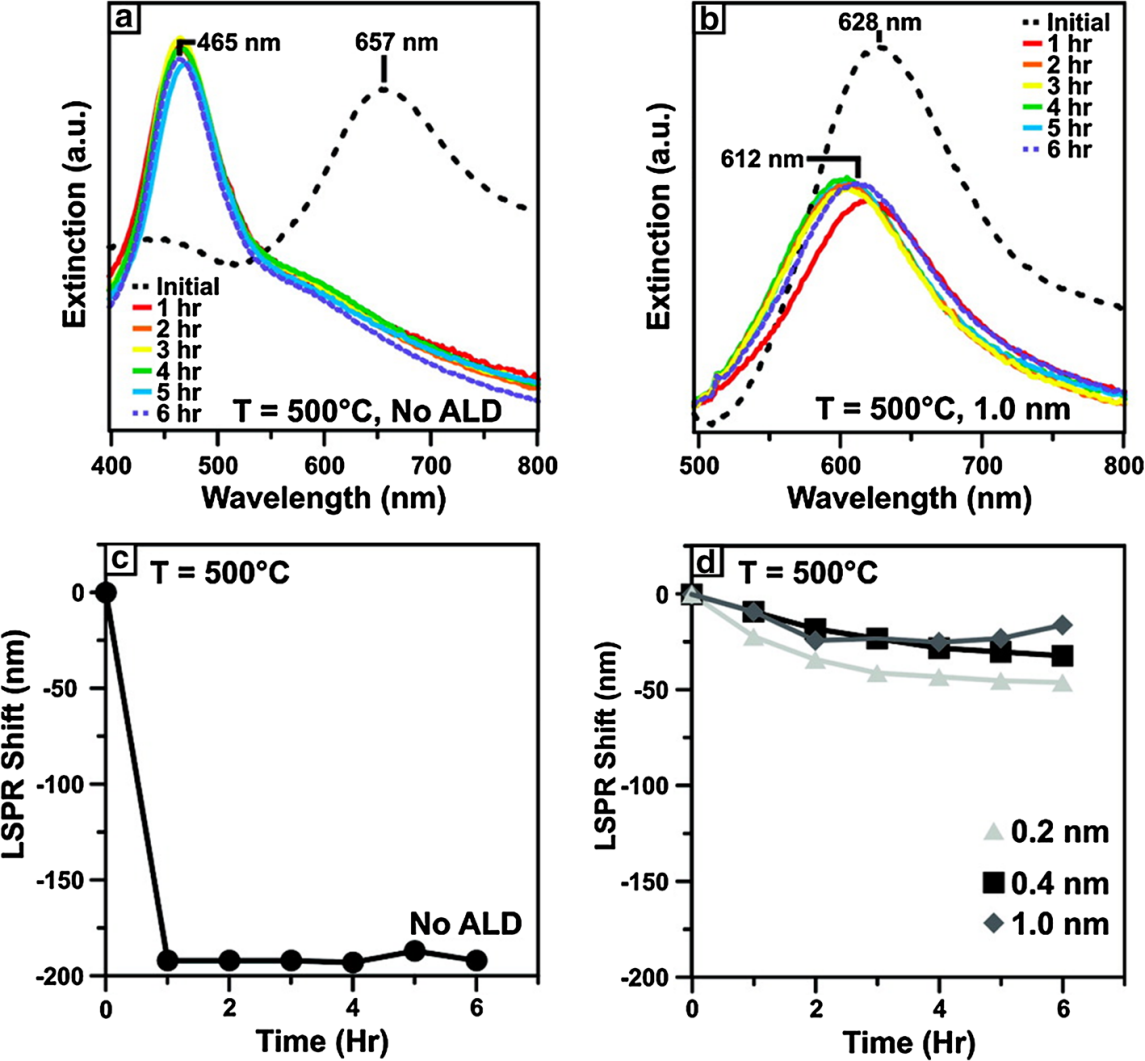
Surface plasmon resonance (SPR) spectroscopy of bare Ag nanoparticles (**a**) and Ag nanoparticles coated with 1.0 nm atomic layer deposition (ALD) Al_2_O_3_ (**b**) heated at 500 °C over time. (**c**) Plot of the SPR shift of Ag nanoparticles coated with 0.2 nm (*grey triangle*), 0.4 nm (*filled square*), and 1.0 nm (*filled diamond*) ALD Al_2_O_3_ heated at 500 °C over time. All samples were heated under 1 Torr N_2_. Reproduced with permission from Ref. [7]. Copyright American Chemical Society, 2007

The temperature dependence of SERS with metal nanoparticles, while significantly influenced by sintering, is also highly dependent on the effect of heating on the surface plasmon resonance (SPR) of the metal nanoparticles, and its response under heating. Several research groups have found that the SPR bands for SERS substrates can red-shift upon heating, with the effect being more dramatic upon nanoparticles that planar substrates [97, 98]. Research work in the Van Duyne group at Northwestern University discovered that using atomic layer deposition (ALD) to apply a thin alumina coating over the SERS substrate significantly increases their thermal stability, the impressive results of which are illustrated in Fig. 10 [7]. Naturally a thicker coating leads to enhanced thermal stability, though coatings of as little as 0.2 nm alumina led to a substantial increase in stability. It is important to mention here that SHINERS have the potential to extend this notion of thermal stability to SERS nanoparticles [6].

### Spectral Reproducibility and Interpretation

The lack of measurement reproducibility is a major limitation of SERS measurements that must be taken into consideration in all measurements, and is in no small way also related to the surface chemistry and related stability of SERS substrates. A lack of substrate generality is intrinsic to the SERS mechanism, as one of the major SERS mechanisms—the chemical enhancement mechanism—works via a charge transfer mechanism. Therefore, if an analyte is not chemically compatible with the surface charge of the SERS substrate, signal enhancement is unlikely to occur.

SERS substrates themselves must also be reproducible synthetically for the results to be truly reproducible. Highly-ordered planar substrates are known to give the most regular SERS spectra, however they sacrifice the high spectral intensity seen when SERS nanoparticles form so-called ‘hot-spots’. The use of multivariate data analysis of spectral variance in SERS measurements using colloids is one potential method to untangle some of the variance seen in SERS measurements. The method takes into account the variation of SERS between different batches of colloids, as the synthesis method may not have been reproduced always exactly [99]. This observation also implies that results may differ from one laboratory to another, and even within the same laboratory variations in time may occur as not always the same persons are in charge of synthesizing the SERS substrates or SERS-active nanoparticles.

Combination of techniques, already employed frequently in heterogeneous catalysis research, may shed further light on the interpretation of SERS measurements. For example, an integrated AFM—Raman instrument is able to directly correlate single nanoparticles or clusters of nanoparticles to spectra, and so give further insight into the chemistry occurring on SERS substrates. The integration of AFM with Raman spectroscopy has been recently illustrated by Harvey et al. for the photo-degradation of rhodamine-6G in combination with differently sized and shaped Ag nanoparticles in the presence and absence of air and at elevated temperatures by making use of an in situ reaction chamber [100].

## Showcase SERS and TERS Studies from Our Laboratory

Recent SERS and TERS work from our group has focused on two photo-catalytic/plasmon-driven catalytic reactions involving two thiophenols, namely pNTP and pATP. Both photo-reactive molecules have been largely studied in the literature, as discussed before, as their reactivity can be tuned by altering the laser excitation wavelength as well as power. In other words, the reduction processes of pNTP and pATP can be regarded as ideal model reactions for the (further) development of the SERS and TERS spectroscopic toolkit for heterogeneous catalysis research.

### SERS of the Photo-catalytic Reduction of pNTP in a Self-assembled Monolayer

A large discussion point in the literature is the interpretation of the reaction product SERS spectrum during the photo-catalytic reduction of pNTP. There exist mainly two proposals: *p*-aminothiophenol (pATP) and *p*,*p*′-dimercaptoazobisbenzene (DMAB). The challenge in the identification of the reaction product is at least twofold: (1) an extremely low number of molecules that are converted during the photo-catalytic reduction reaction, and (2) the few molecules present or formed are chemically bound to the surface, and therefore non-accessible by other characterization methods with molecular fingerprinting capabilities.

**Fig. 11:**
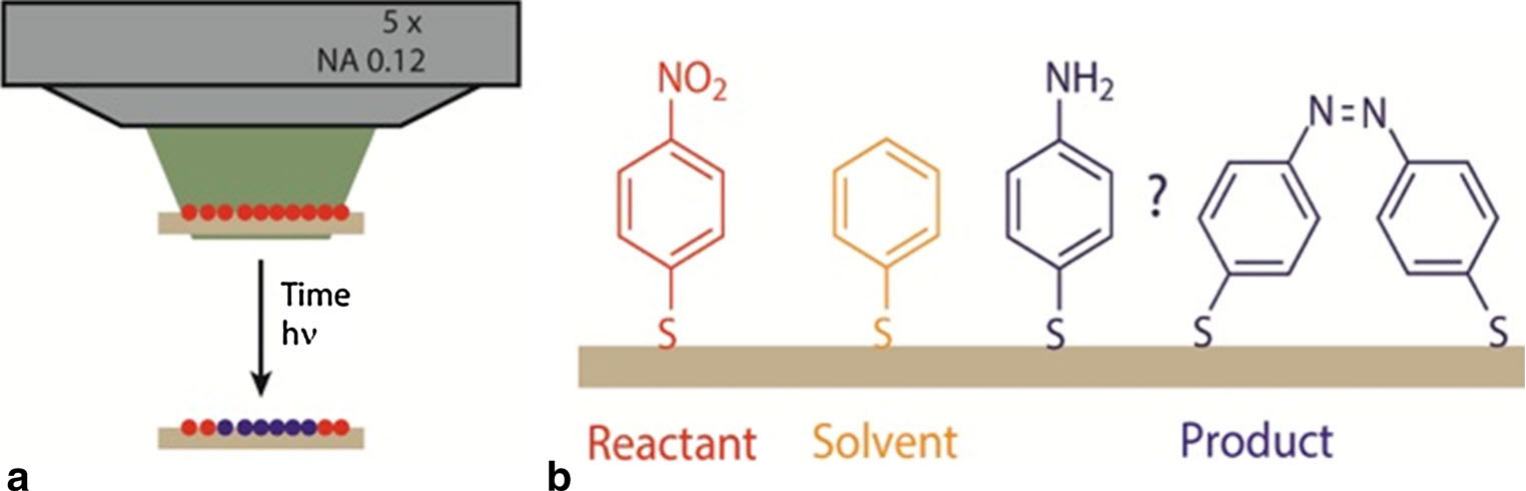
**a** SERS approach on a self-assembled monolayer (SAM) on Ag island films as SERS substrate in which only the photo-catalytic reaction at the surface is studied as a function of the irradiation with a 532 nm laser and (**b**) The photo-reduction of p-nitrothiophenol to either *p*-aminothiophenol or *p*,*p*′-dimercaptoazobisbenzene. For dilution experiments, thiophenol has been used as the 2D-equivalent of a solvent molecule. Reproduced with permission from Ref. [101]. Copyright Wiley–VCH 2014

In an attempt to resolve this issue, Van Schrojenstein Lantman and co-workers have developed a two-dimensional approach making use of a self-assembled monolayer (SAM) of pNTP with Ag island films as SERS substrate to determine the reaction kinetics upon laser irradiation making use of SERS [101]. This approach is outlined in Fig. 11. By following the characteristic Raman peaks of pNTP and the reaction product it was possible to reveal in two distinctly different ways that this photo-catalytic reduction process involves a dimerization reaction, pointing towards the assignment of the reaction product as DMAB.

**Fig. 12:**
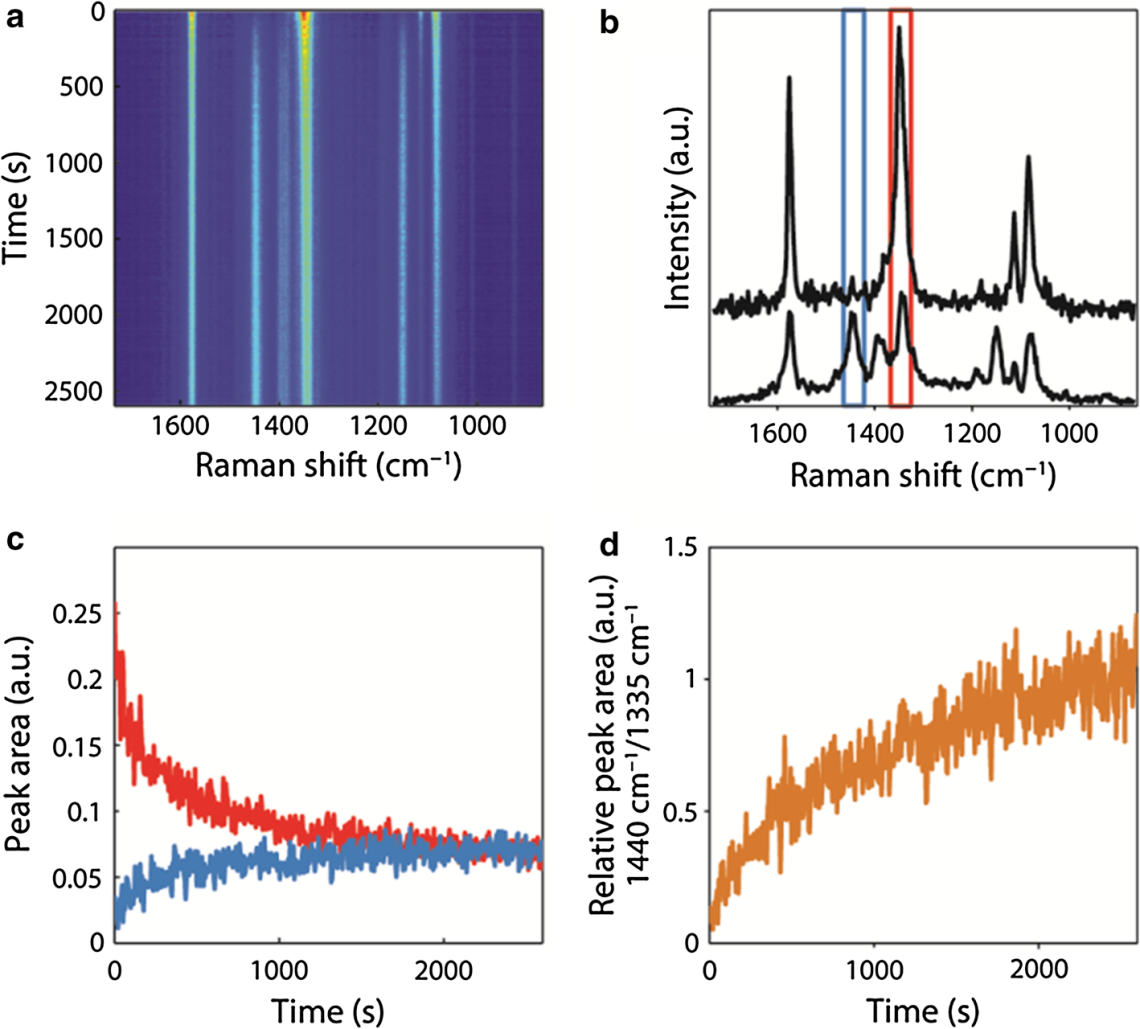
**a** Time-dependent SERS measurements at 532 nm laser irradiation from a pNTP-coated Ag island film, each *horizontal line* represents a (*colour-coded*) spectrum. **b** The first (*top*) and last (*bottom*) spectrum of (**a**). The *coloured bands* depict peak integration area for quantification of pNTP (1293–1373 cm^−1^ and product (1,412–1,473 cm^−1^) in time. **c** Time traces for pNTP (*red*) and product (*blue*) as taken from peak areas shown in (**b**). **d** Relative peak area as function of time. Reproduced with permission from Ref. [101]. Copyright Wiley–VCH 2014

The first approach involved the determination of the reaction kinetics, as illustrated in Fig. 12. The two characteristic Raman peaks for tracking the reaction are 1,335 cm^−1^ (reactant pNTP, asymmetric NO_2_-stretch) [22] and 1,440 cm^−1^ (product, assigned to pATP as a b_2_-mode [23], or to DMAB as a N=N stretch) [13a]. Figure 12 shows that it is possible to obtain an idea of the surface concentration of the reactant and reaction product by tracking the peak areas of both species as a function of time upon green laser light irradiation. It was found that the concentration of pNTP decays in time, while synchronously the reaction product of the photo-catalytic process is formed. It was postulated that the reaction mechanism [24] has a single rate-determining step and consequently the reaction was expected to be a one-step process. In this manner, the reaction order could be determined for the rate-limiting step and turned out to be two.

The second approach providing evidence for a dimerization reaction of pNTP was obtained through incorporation of an inert thiol into the SAMs of pNTP, as illustrated in Fig. 11b. Thiophenol was here used as the two-dimensional equivalent of a solvent. It was found that the reaction rate was extremely slowed down by the dilution effect with thiophenol, up to the point that no photo-reduction reaction was observed under the standard reaction conditions applied. As a result, the reduction process of pNTP should be second-order.

### SHINERS Studies of the Photo-catalytic Reduction of pATP

Harvey and co-workers have recently assessed the performance of SHINERS for investigating the photo-reactivity of the anticipated reaction intermediate in the process of the photo-catalytic reduction of pNTP, i.e., pATP [102]. It is known that pATP, as pNTP, interacts strongly with either Ag and Au, giving strong SERS signals. As mentioned before, analogous to pNTP also pATP is regarded a model compound for probing SERS activity. However, it was noted before that extra Raman bands form in the SERS spectra with an excitation wavelength of 633 nm or below. Difficulties in producing the SERS spectra of pure pATP led to detailed investigations of this effect, from which two potential explanations were derived. The first explanation involves the catalytic conversion of pATP into DMAB, which is then considered to be the molecule responsible for the changes in the SERS spectra of pATP [102]. This line of thinking is supported by further theoretical and experimental work, as discussed in detail above and is regarded as a plasmon-driven reaction, similar pNTP. Another explanation has been put forward by Kim et al., suggesting that the SERS bands under debate are simply a result of charge transfer, and therefore a reversible chemical enhancement [87–89].

**Fig. 13:**
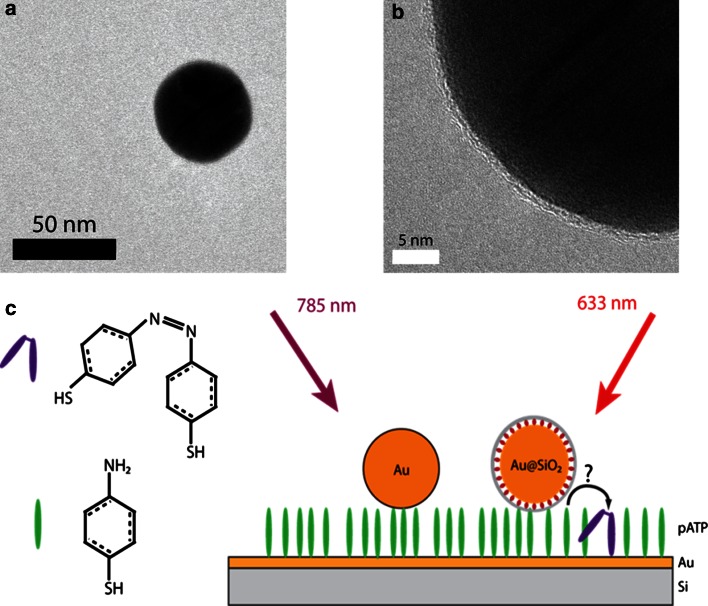
(**a**) and (**b**) High-resolution transmission electron microscopy (HRTEM) images of a SHINER, consisting of a silica-coated Au nanoparticle; and **c** Schematic of a self-assembled monolayer (SAM) of pATP on gold on a silicon substrate. Nanoparticles, either gold nanoparticles or gold-based SHINERS, are deposited on top of this pATP monolayer. The sample is then irradiated with a 785 nm laser excitation first, followed by an irradiation with a 633 nm laser excitation. After Ref. [102]

**Fig. 14:**
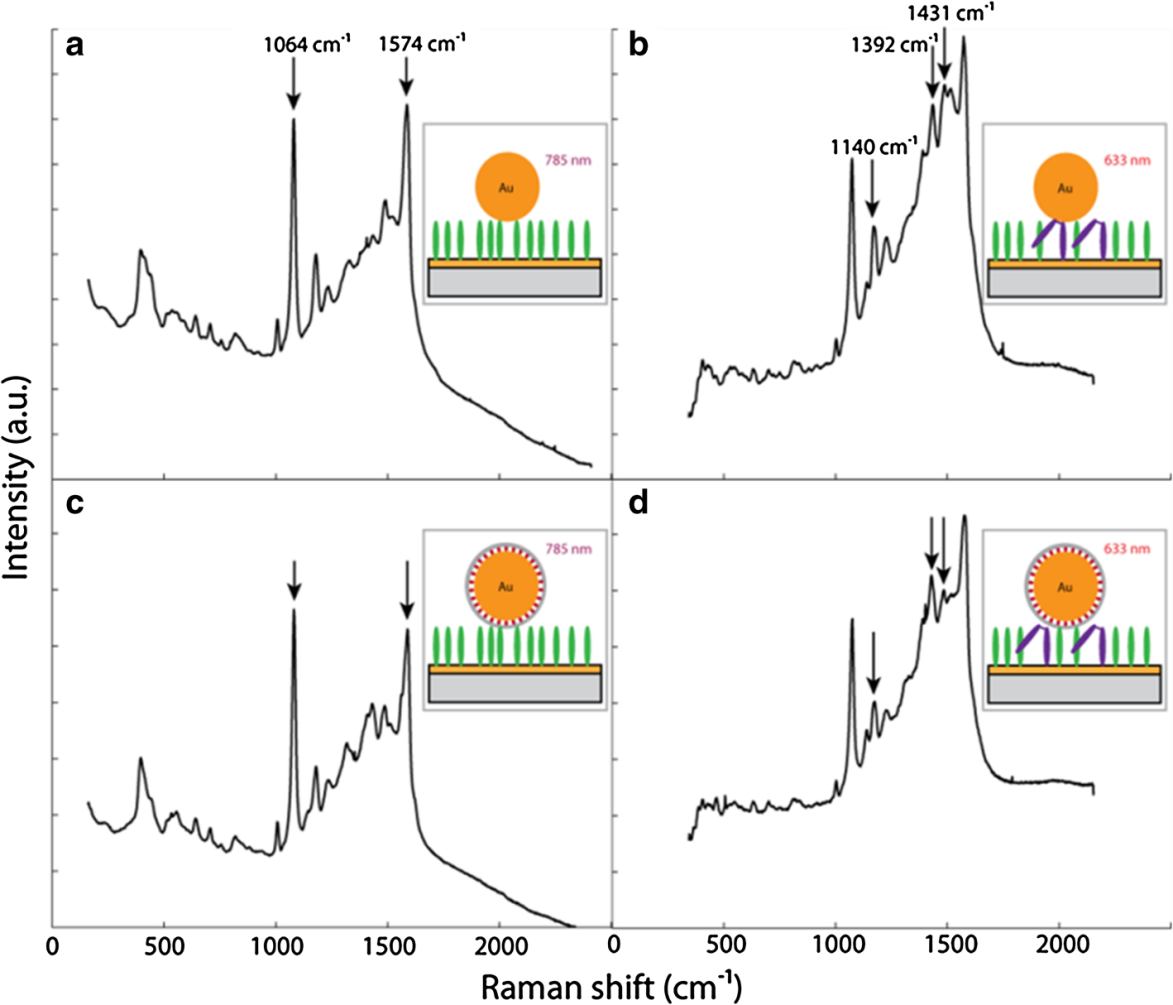
Raman spectra of a surface layer of pATP in contact with bare gold nanoparticles after exposure to (**a**) 785 nm laser light; (**b**) 633 nm laser light; Raman spectra of a surface layer of pATP in contact with gold-based SHINERS after exposure to (**c**) 785 nm laser light; and (**d**) 633 nm laser light. After Ref. [102]

In order to shed further insights in both hypotheses, Harvey et al. have investigated the behaviour of pATP under laser light irradiation in the presence of both bare gold nanoparticles and Au-based SHINERS [102]. This was done using a 785 nm laser excitation to record the initial pATP spectra, and a 633 nm laser excitation to trigger the spectral changes in the Raman spectra. The methodology used is outlined in Fig. 13, while the obtained results are summarized in Fig. 14. Theoretically, if a charge transfer process through the Au nanoparticles is the cause of the unexpected spectral bands at 1140, 1392 and 1431 cm^−1^, these Raman bands will be observed over uncoated gold nanoparticles, but not over the Au-based SHINERS. To obtain sufficient statistics maps of 2,000 spectra per sample were averaged using Matlab to give the average spectra shown in Fig. 14.

The dominance of the bands at 1064 and 1574 cm^−1^ in the Raman spectra in Fig. 14a and c are indicative of the presence of pATP, as exposure to 785 nm laser light results only in extremely slow conversion of the starting material into DMAB. The spectra in Fig. 14b and d, however, show clear Raman bands at 1140, 1392 and 1431 cm^−1^, indicative of the conversion of pATP over both bare gold nanoparticles and Au-based SHINERS. The reason that no complete conversion is observed could be linked to an equilibrium state, and/or also to the fact that only a small fraction of the observed molecules contribute to this signal. The observation of the spectral changes over both bare gold nanoparticles and Au-based SHINERS can be seen as a confirmation that there is a photo-catalytic reduction of pATP into DMAB as charge transfer cannot take place through the oxidic and isolating shell surrounding the Au nanoparticles, as illustrated in Fig. 13b by the HRTEM of the Au-based SHINERS. This observation provides further evidence that the hypothesis of Kim et al. [87–89] is rather unlikely to occur and therefore we conclude that the origin of the spectral changes is due to the formation of DMAB as the dimerization product of pNTP, as has also been supported by theoretical and experimental work by Sun et al. [81, 84, 85].

### TERS Studies of the Photo-catalytic Reduction of pNTP

**Fig. 15:**
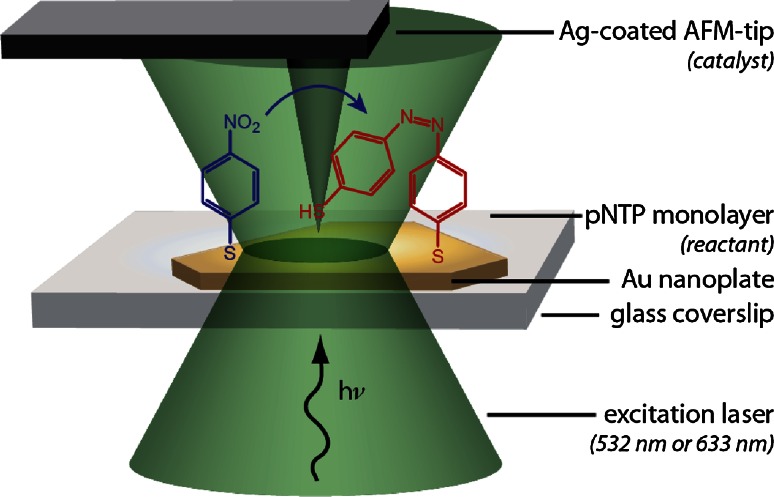
React-analyse TERS for monitoring the photo-catalytic reduction of pNTP to DMAB making use of a dual laser excitation approach. TERS spectra are measured making use of 633 nm laser excitation, while the chemical reaction is triggered by a 532 nm laser when the Ag-coated AFM tip is in contact with the pNTP monolayer. Redrawn with permission from Ref. [103]. Copyright Nature Publishing Company 2012

In a recent study Van Schrojenstein Lantman and co-workers reported on the first stepwise ‘react-analyse’ application of time-resolved TERS [103]. This unique TERS approach is schematically illustrated in Fig. 15. Here, the photo-catalytic reduction process of pNTP to DMAB has been monitored by employing two different excitation wavelengths: i.e., a green laser (532 nm) to induce the photo-reaction, while a red laser (633 nm) was used to probe the transformation process during the reduction reaction with Raman spectroscopy. By exploiting this multi-probe TERS methodology, it became possible to follow the catalytic activity of a Ag-coated TERS-tip. By making use of a SAM of pNTP on a Au nanoplate it was ensured that no more than a monolayer of reactant is studied. As only the utmost point of the TERS tip is in contact with the reactant, one can consider this is a first of its kind study of a single catalyst ‘particle’ in action.

**Fig. 16:**
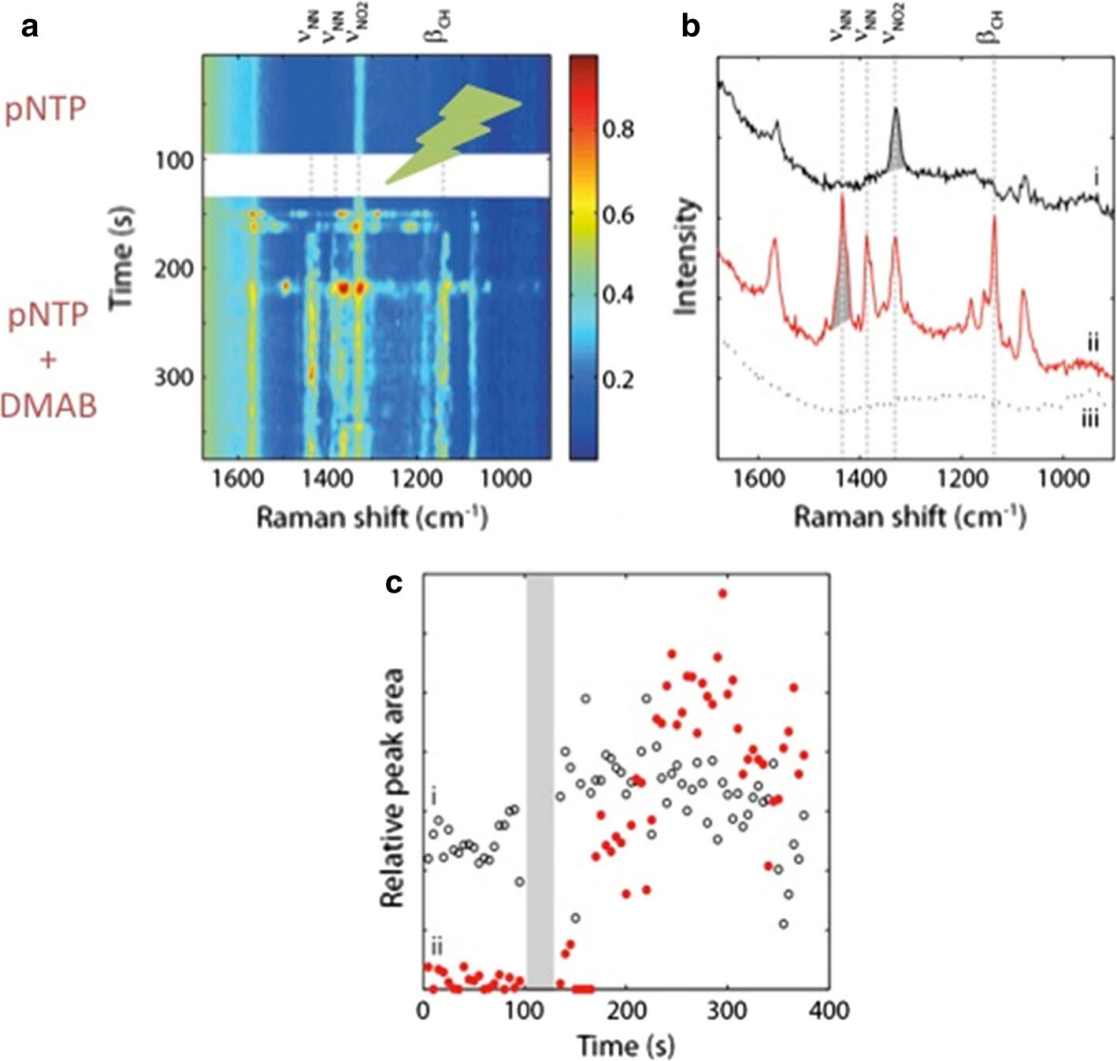
**a** Time-dependent TERS spectra at 633 nm excitation (5 s integration time, 380 µW) shown before (*top*) and after (*below white band*) illumination; **b** two spectra from a are shown: spectrum (*i*) is taken at 90 s and spectrum (*ii*) at 265 s. Spectrum (*iii*) is the reference spectrum taken after the time-dependent spectra. *Asterisks* in (**a**) and (**b**) indicate the location of the 950 cm^−1^ band of the SiO_2_–glass signal of the glass substrate; **c** *peak areas* as a function of time for the pNTP band at 1,335 cm^−1^ (*i*) and for the band at 1,440 cm^−1^ (*ii*), belonging to DMAB. The period of green illumination between 100 and 130 s is indicated by the *shaded band*. Reproduced with permission from Ref. [103]. Copyright Nature Publishing Company 2012

The combination of green and red laser sources was found to be crucial for controlled observation of the photo-catalytic reaction of pNTP to DMAB at the catalytic site, as shown in Fig. 16 [103]. Upon green laser excitation the photo-catalytic reduction reaction can be temporarily activated and the typical Raman bands of pNTP and DMAB at around 1,335 and 1,440 cm^−1^ can be followed as a function of time during this reduction process at the nanoscale.

## Future Perspectives

With significant advances being made rapidly throughout the SERS community, the prospect of performing operando SERS and TERS measurements of a catalytic reaction are clearly on the horizon. The introduction and related versatility of SHINERS in the spectroscopic toolkit is an important step forward along the way to introducing the SERS method as a core analysis technique for heterogeneous catalysis research.

**Fig. 17:**
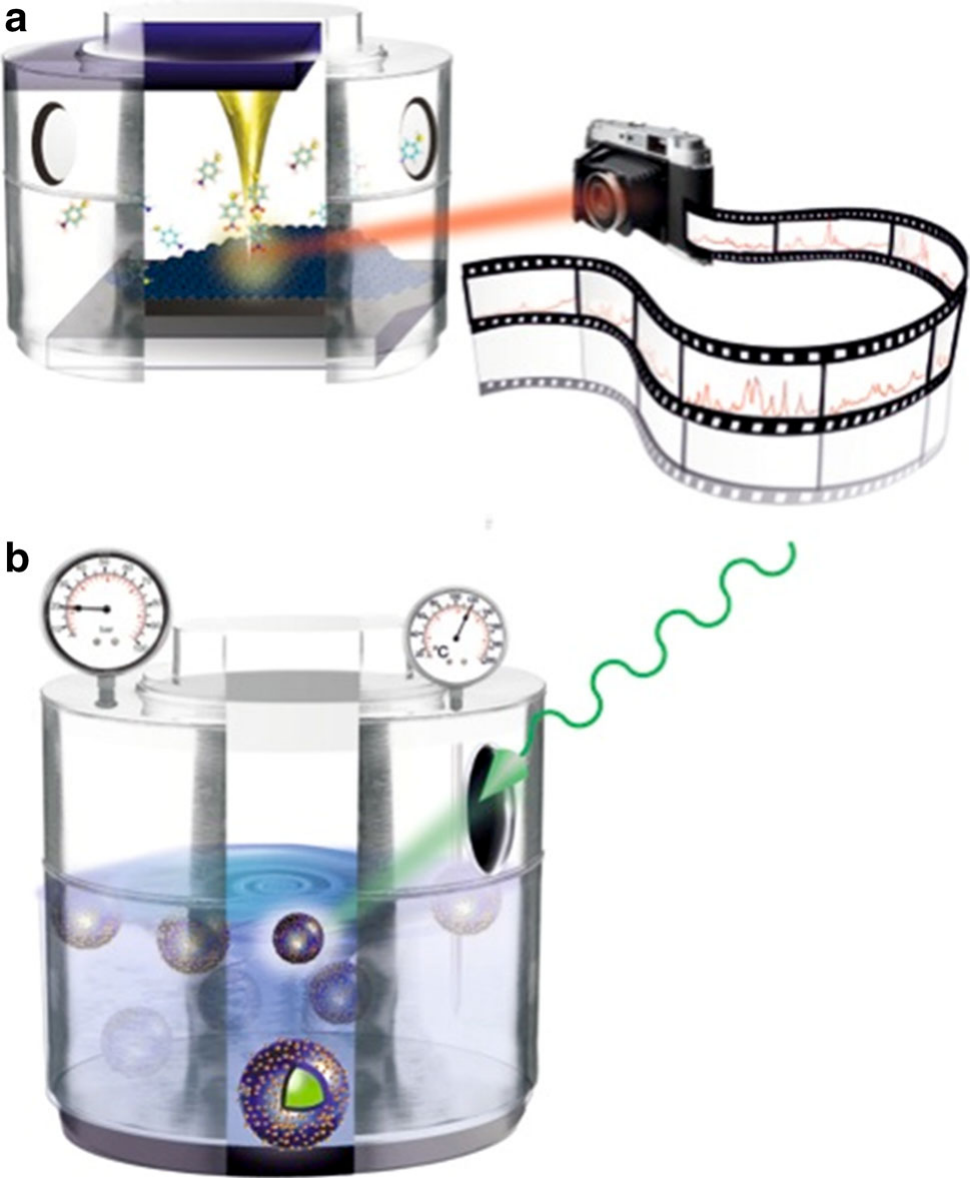
**a** Schematic of an operando TERS set-up operating at elevated reaction temperature and pressure allowing to trace reactants, reaction products, as well as reaction intermediates during a catalytic process at the gas–solid interface; and **b** Schematic of an operando SERS set-up operating at elevated reaction temperature and pressure making use of SHINERS to monitor the reactants, reaction products as well as reation intermediates during a catalytic process at the liquid–solid interface

Already in recent times a couple of preliminary studies for utilizing tip-enhanced Raman spectroscopy (TERS) in catalysis have demonstrated its potential [104, 105]. As shown in one of the showcase examples above, Van Schrojenstein Lantman and co-workers used TERS to follow a photo-activated catalytic reaction on the nanoscale, and research is ongoing towards single molecule TERS sensitivity and of improving the spatial resolution of this optical technique into the nanometer scale, as well as expanding the measurement conditions towards more realistic ones [103]. Such operando TERS approach is schematically shown in Fig. 17a.

The advent of surface-enhanced spatially-offset Raman spectroscopy (SESORS), thus far mainly applied to biological systems, also demonstrates great potential for application in catalysis [106, 107]. Perhaps the most significant challenge in the full applicability of SERS as a standard spectroscopic tool for catalysis research is furthering the work done thus far on the chemical generality or SERS, developing either a single method or a whole toolkit of SERS substrates that enable the detection of any analyte, irrespective of charge or hydrophobicity/hydrophilicity. The first steps have been taken along this promising path, with the initial development of transition metals-coated substrates, and more recently the invention of SHINERS, the first SERS substrate to truly separate heterogeneous catalysis and spectroscopic analysis (reaction monitoring). [19, 108] SHINERS could really become an asset for monitoring the reactants, reaction products, as well as minute amounts of reaction intermediates, in a reactor vessel, as illustrated in Fig. 17b. The showcase example of Heck and co-workers is a clear indication that this indeed may become possible. Moreover, more combinations of SHINERS and reactors and (individual) catalyst particles and catalyst bodies can be envisaged, providing local “Raman antenna” for catalytic performance, including activity, selectivity as well as stability.
